# Development of a deep learning-based prediction model for postoperative delirium using intraoperative electroencephalogram in adults

**DOI:** 10.1038/s41746-025-02033-y

**Published:** 2025-11-17

**Authors:** Jang Ho Ahn, Hyeonhoon Lee, Pedro Gambus, Hyun-Kyu Yoon, Jae-Woo Ju, Hyung-Chul Lee

**Affiliations:** 1 Department of Anesthesiology and Pain Medicine, Seoul National University College of Medicine, Seoul National University Hospital, Seoul, South Korea; 2 Healthcare AI Research Institute, Seoul National University Hospital, Seoul, South Korea; 3 Department of Medicine, Seoul National University College of Medicine, Seoul, South Korea; 4 Department of Transdisciplinary Medicine, Seoul National University Hospital, Seoul, South Korea; 5 Anesthesiology Department, Hospital Clinic de Barcelona, and Institut d’Investigacions Biomèdiques Agusti Pi i Sunyer (IDIBAPS), Barcelona, Spain

**Keywords:** Machine learning, Risk factors

## Abstract

Postoperative delirium (POD) is associated with increased morbidity and mortality. This study aims to develop a deep learning-based model (DELPHI-EEG) to predict postoperative delirium using intraoperative electroencephalogram (EEG) waveform. A total of 34,550 surgical cases (267 event cases), with 6-lead intraoperative EEG monitoring between 2022 and 2024, were included for model development. During 5-fold cross-validation, the DELPHI-EEG model showed an area under the receiver operating characteristic (AUROC) curve of 0.870 (95% confidence interval [CI]: 0.789–0.935) and the area under the precision-recall curve (AUPRC) of 0.038 (95% CI: 0.017–0.084), significantly outperforming the logistic regression model using burst suppression ratio with AUROC of 0.729 (95% CI: 0.624–0.825, *p* = 0.004) and AUPRC of 0.013 (95% CI: 0.007–0.026, *p* = 0.002). The DELPHI-EEG model might serve as a risk predictor for postoperative delirium, potentially enabling targeted preventive interventions for surgical patients; nonetheless, external validation in diverse clinical settings is required.

## Introduction

Postoperative delirium (POD) is an acute neuropsychiatric syndrome characterized by an acute onset of impaired awareness, attention, and cognition after surgery^1^. With an incidence ranging from 10% to 60% depending on the surgical population and an average time to onset of delirium of 2.1 ± 0.9 days, POD can lead to increased 5-year mortality (odds ratio = 7.35; 95% CI = 1.49–36.18), unplanned intensive care unit admission, prolonged hospital stay, discharge to non-home settings, decline in activities of daily living, and higher healthcare costs^2–5^. Additionally, sociodemographic disparities—including lower educational attainment and neighborhood socioeconomic disadvantage—are associated with increased POD risk^6^. Therefore, developing accurate and accessible predictive models is essential for mitigating these disparities by supporting timely interventions in vulnerable groups.

Despite its significant clinical impact, early prediction of POD remains challenging. Current delirium risk prediction tools primarily rely on preoperative factors, such as age, cognitive function, physical health, and medical history^2,7,8^. Although such tools provide valuable risk stratification before surgery, most of them cannot account for intraoperative physiological changes that may contribute to POD development. Some intraoperative monitoring techniques, such as transcranial Doppler and near-infrared spectroscopy, have been used for POD risk assessment^8^. However, these methods require additional equipment, increase costs, and only evaluate the cerebral blood flow in limited brain regions. Given these limitations, there is an urgent clinical need for accurate, intraoperatively available tools that can identify patients at high risk for POD early, allowing for timely preventive strategies and targeted postoperative care.

Electroencephalogram (EEG) monitoring is routinely performed during surgery under general anesthesia and provides continuous assessment of brain function. Prior studies have shown that intraoperative EEG parameters, such as burst suppression or changes in delta, theta, or fast frequency activity during procedures, are correlated with POD incidence^9,10^. In particular, the presence of EEG burst suppression has been reported as a significant predictor of POD, with patients who developed POD showing a mean burst suppression duration increase of 15.86 min compared to that in those without POD^11^. However, these models exclude other EEG features that showed significant differences between the patients with and without POD, such as alpha, delta, and theta power, highlighting the need for further analysis to explore the potential of these additional EEG features^9,12,13^.

With the advancement of deep learning techniques, a growing number of studies have applied EEG signals to various clinical tasks, such as diagnosis and prediction, including intraoperative hypotension prediction using biosignal waveforms^14–16^. In a previous study using 10-lead EEG signals, a vision transformer-based model predicted delirium in mechanically ventilated critically ill patients with 97% test accuracy^17^. Machine learning (ML) models combining handcrafted features of intraoperative frontal EEG and clinical parameters have been developed for POD prediction and have achieved areas under the receiver operating characteristic (AUROC) curve values of 0.887 and 0.77, respectively^18,19^. However, no study has been conducted on predicting postoperative delirium using deep-learning techniques with multi-channel intraoperative EEG waveforms.

Recently, a deep learning-based spatiotemporal encoding framework has been proposed based on resting state EEG to predict postoperative cognitive function^20^. This architecture combines a graph convolutional network to capture connectivity patterns between brain regions, a convolutional neural network to extract hierarchical spatial and temporal features, and a Transformer module to model dependencies across time points. Such a framework is particularly promising for predicting POD due to POD’s association with multi-level EEG alterations, including alpha power and frontoparietal alpha coherence, and connectivity between the occipitoparietal and frontal cortex^21–23^. Therefore, we adapted this spatiotemporal encoding framework to utilize intraoperative EEG for POD prediction. EEG features other than burst suppression would be utilizable if the multi-channel EEG signals are input into the model.

The primary objective of our study was to develop a DEep Learning-based model for POD Hazard assessment using Intraoperative EEG (DELPHI-EEG). We compared the predictive performance of DELPHI-EEG with that of a conventional logistic regression model and ML models incorporating the burst suppression ratio (SR), Patient State Index (PSI), age, and sex. We hypothesized that DELPHI-EEG would capture various EEG features beyond burst suppression, thereby demonstrating its potential as an artificial intelligence-assistant tool for POD prediction in surgical patients who underwent general anesthesia. The primary endpoint of our study was the occurrence of POD, defined according to psychiatric consultations and/or administration of delirium-related antipsychotics.

## Results

### Study population

**Fig. 1:**
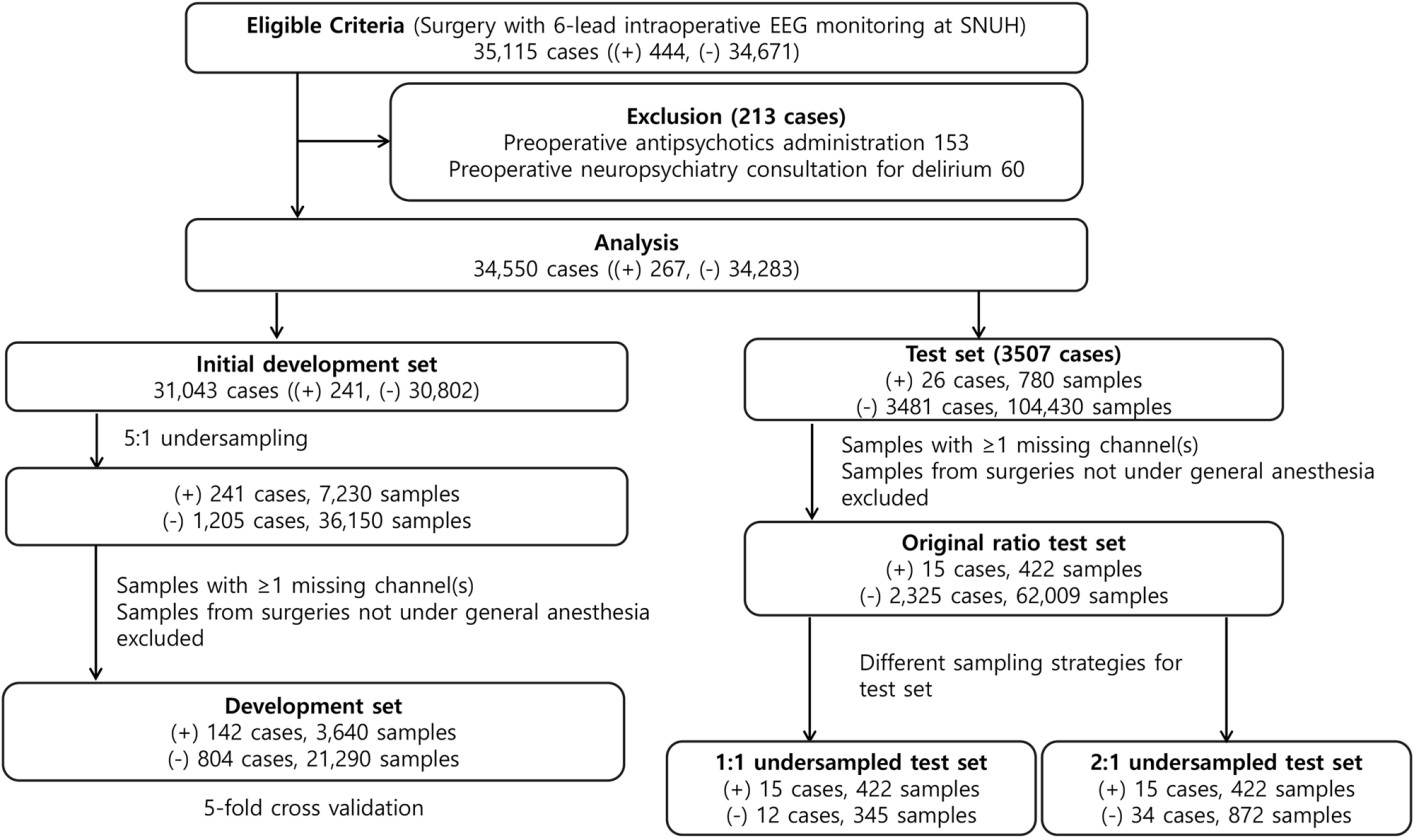
Inclusion and exclusion criteria, and the number of cases and samples for the development and test sets. EEG electroencephalography, SNUH Seoul National University Hospital, NP neuropsychiatry, ( + ), positive labels for postoperative delirium; (−), negative labels for postoperative delirium.

A total of 35,115 surgical cases were identified, comprising 34,671 negative and 444 positive cases (Fig. 1). The dataset was randomly split by unique patients into a development set (30,802 negative and 241 positive cases) and a test set (3481 negative and 26 positive cases). The development set was further undersampled randomly to 1205 negative and 241 positive cases. Sample exclusion due to missing EEG channels or procedures not involving general anesthesia resulted in 21,290 negative and 3640 positive samples (804 negative and 142 positive cases) in the development set, and 62,009 negative and 422 positive samples (2325 negative and 15 positive cases) in the test set. The test set was evaluated across three sampling strategies: an original distribution set (2325 negative and 15 positive cases), a 1:1 undersampled set (12 negative and 15 positive cases), and a 2:1 undersampled set (34 negative and 15 positive cases). The mean age of the analyzed cases in the development and test set was 58.72 ± 15.44 years (58.66 ± 15.46 years in the development set; 59.22 ± 15.31 years in the test set), and the proportion of males was 43.3% (43.5% in the development set; 43.3% in the test set). Among the POD-positive cases, the median time from surgery to POD onset was 4 days (Q1: 2 days, Q3: 12 days).

### Comparison of model performance

**Fig. 2:**
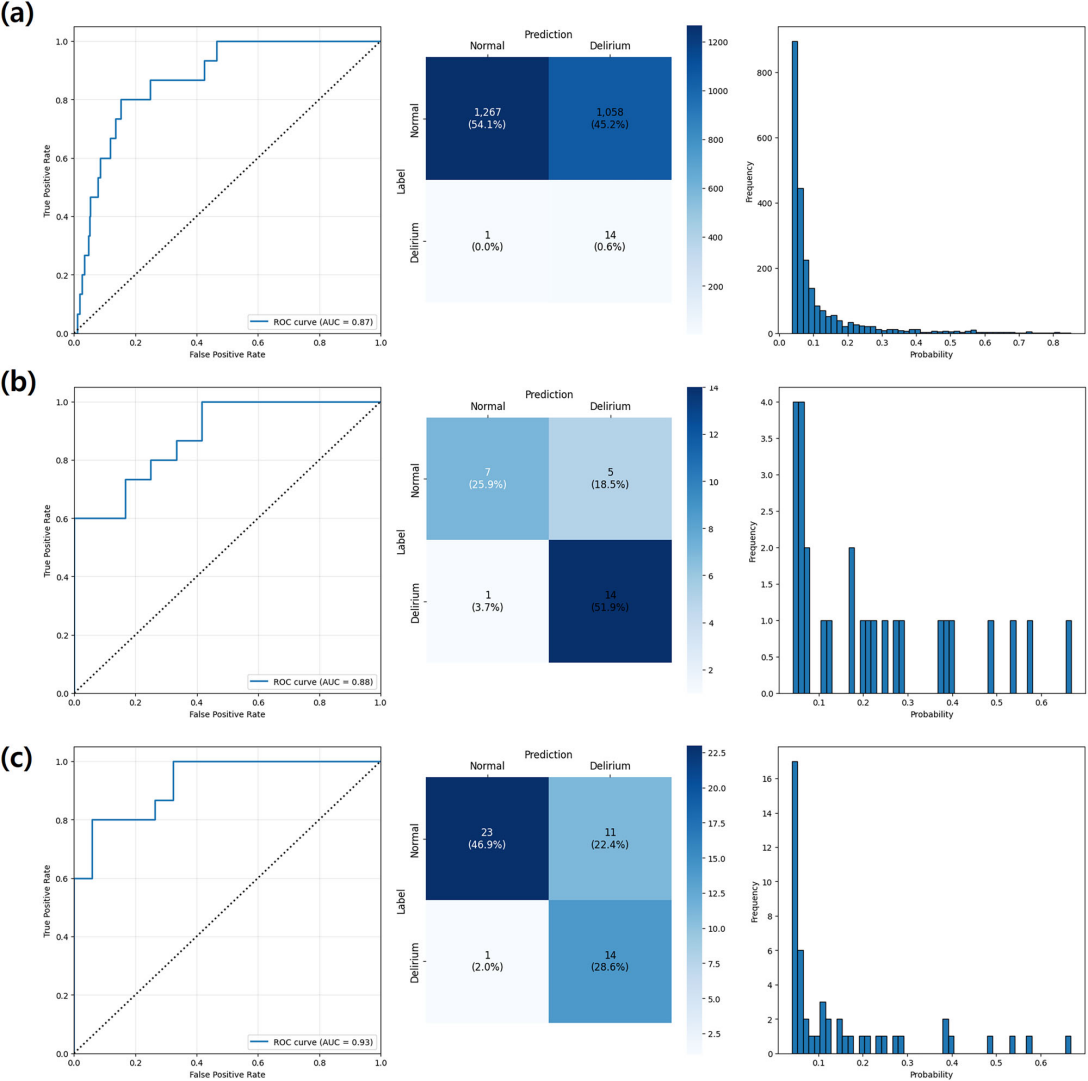
The receiver operating characteristics curve, confusion matrix, and predicted probability histogram of DELPHI-EEG for different test set sampling strategies. **a** 155:1 (original ratio), **b** 1:1 undersampled, and **c** 2:1 undersampled test sets.

**Fig. 3:**
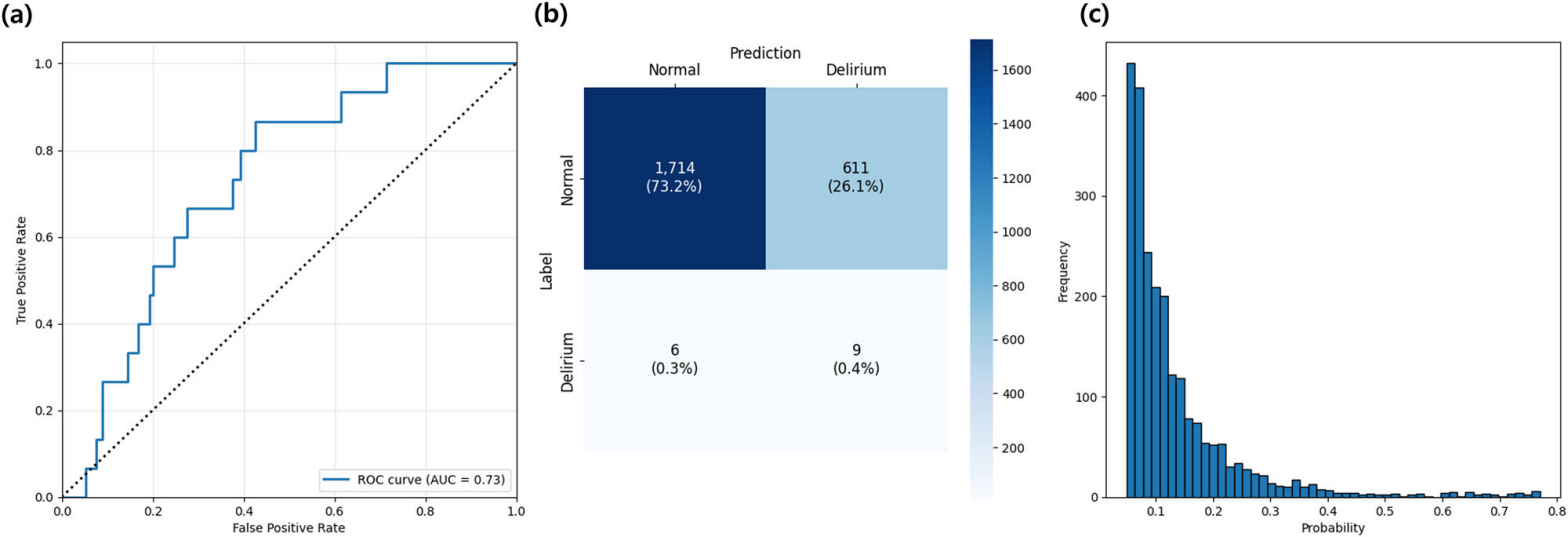
Performance of the logistic regression model in the 155:1 test set. **a** The receiver operating characteristic curve, **b** confusion matrix, and **c** predicted probability histogram.

Figures 2 and 3 show that the AUROC of the DELPHI-EEG was 0.870 (95% confidence interval [CI]: 0.789–0.935) (Fig. 2a), outperforming the baseline logistic regression model based on the duration of time where SR > 1%, area under the SR versus time curve where SR > 1%, duration of time where PSI < 25, age, and sex (AUROC: 0.729 [95% CI: 0.624–0.825]) in the testing set with the original ratio (*p* = 0.004) (Fig. 3a). The performances of ML models based on the same input features including XGBoost (XGB), LightGBM (LGB), Random Forest (RF), and Gradient Boosting Classifier (GB) were an AUROC of 0.801 (95% CI: 0.673–0.891), 0.798 (95% CI: 0.675–0.890), 0.799 (95% CI: 0.660–0.901), and 0.764 (95% CI: 0.620–0.875), respectively. The other evaluation metrics, including the area under the precision-recall curve (AUPRC), F1 score, accuracy, Brier score, integrated calibration index (ICI), precision, and recall, are presented in Table 1 and Supplementary Table 1. The AUPRC and the recall of DELPHI-EEG were 0.038 (95% CI: 0.013–0.040) and 0.933 (95% CI: 0.778–1.000), respectively.

**Table 1:** The evaluation metrics of different models of the 155:1 test set

Models	AUROC [95% CI]	AUPRC [95% CI]	F1 score [95% CI]	Accuracy [95% CI]	Brier score [95% CI]	ICI [95% CI]	Precision [95% CI]	Recall [95% CI]
DELPHI-EEG	0.870 [0.789,0.935]	0.038 [0.017,0.084]	0.026 [0.013,0.040]	0.547 [0.527,0.567]	0.031 [0.028,0.035]	0.107 [0.101,0.113]	0.013 [0.007–0.021]	0.933 [0.778–1.000]
Logistic regression	0.729 [0.624–0.825]	0.013 [0.007–0.026]	0.028 [0.012–0.048]	0.736 [0.718–0.754]	0.034 [0.030–0.038]	0.128 [0.122–0.133]	0.015 [0.006–0.025]	0.600 [0.333–0.857]
XGBoost	0.801 [0.673–0.891]	0.042 [0.011–0.183]	0.040 [0.019–0.067]	0.777 [0.759–0.793]	0.041 [0.037–0.045]	0.118 [0.112–0.125]	0.021 [0.009–0.035]	0.733 [0.471–0.941]
LightGBM	0.798 [0.675–0.890]	0.022 [0.011–0.047]	0.038 [0.017–0.061]	0.760 [0.742–0.776]	0.043 [0.038–0.048]	0.110 [0.103–0.117]	0.019 [0.009–0.032]	0.733 [0.467–0.938]
Random Forest	0.799 [0.660–0.901]	0.024 [0.011–0.052]	0.045 [0.021–0.075]	0.802 [0.786–0.817]	0.042 [0.037–0.048]	0.100 [0.094–0.108]	0.023 [0.011–0.039]	0.733 [0.471–0.941]
Gradient Boosted Classifier	0.764 [0.620–0.875]	0.018 [0.009–0.039]	0.046 [0.017–0.080]	0.859 [0.844–0.872]	0.052 [0.045–0.059]	0.082 [0.076–0.092]	0.118 [0.112–0.125]	0.021 [0.009–0.035]

DELPHI-EEG used the EEG waveform, age, and sex as model inputs.

Logistic regression, XGBoost, LightGBM, random forest, and gradient boosted classifier used as inputs the duration of time with suppression ratio (SR) > 1%, area under the SR–time curve where SR > 1%, duration of time with the Patient State Index (PSI) < 25, age, and sex.

*AUROC* area under the receiver operating characteristic curve, *AUPRC* area under the precision-recall curve, *ICI* integrated calibration index, *CI* confidence interval.

### Interpretability analysis

SHAP (SHapley Additive exPlanations) analysis for XGB, LGB, RF, and GB models indicated that old age, male sex, long duration of PSI < 25, SR > 1%, and high AUC of SR > 1% correlated with delirium incidence (Supplementary Fig. 1).

**Fig. 4:**
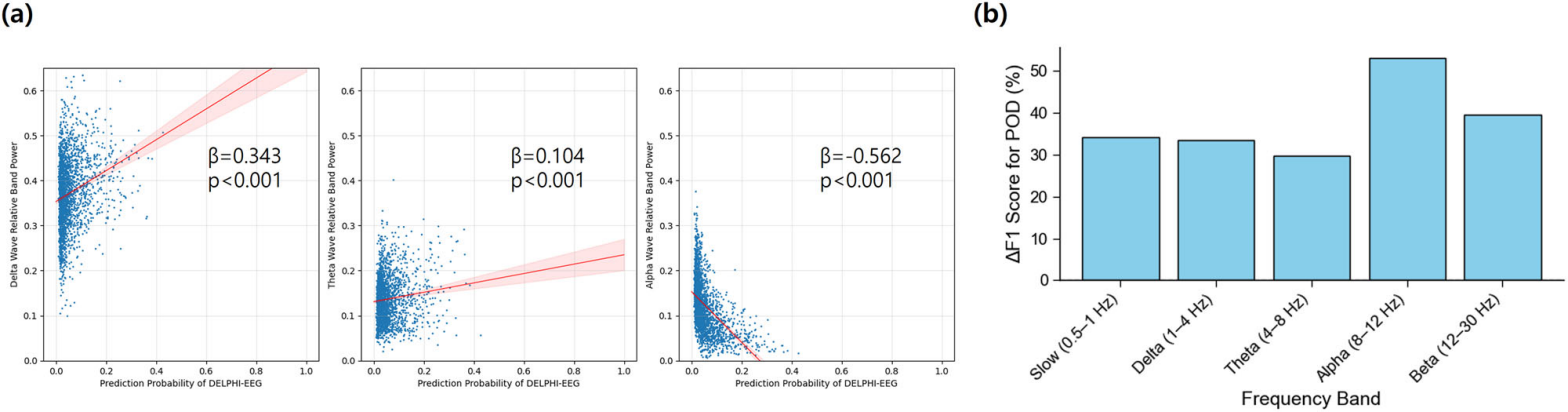
Scatter plots showing the relationship between. **a** DELPHI-EEG predicted probability and relative band power of delta (1–4 Hz), theta (4–8 Hz), and alpha (8–12 Hz) frequency bands. **b** Percent reduction in F1-score for postoperative delirium (POD) prediction following targeted ablation applied to each 2 min sample. *β* denotes the slope from Spearman’s rank correlation test, and *p* denotes the corresponding *p*-value.

Linear regression analysis showed significant associations between DELPHI-EEG predicted probabilities and both the duration of time where SR > 1% and the area under the SR versus time curve where SR > 1%. The regression slopes were 2.112 × 10^3^ (95% CI: 1.890–2.335 × 10^3^) and 3.278 × 10^2^ (95% CI: 2.805–3.750 × 10^2^) respectively for the duration of time where SR > 1% and the area under the SR versus time curve where SR > 1% (all *p* < 0.001) (Supplementary Fig. 2). There were significant associations between DELPHI-EEG predicted probabilities and relative band power in the alpha, delta, and theta frequency ranges. The regression slopes were 0.343 (95% CI: 0.285–0.400), 0.104 (95% CI: 0.068–0.141), and −0.562 (95% CI: −0.601 to −0.523) for the delta, theta, and alpha band powers, respectively (all *p* < 0.001) (Fig. 4a). However, there was no significant association between DELPHI-EEG predicted probabilities and the time to POD onset. The regression slopes were −0.012 (95% CI: −0.027–0.004) (*p* = 0.222) (Supplementary Fig. 3b).

Ablation of individual frequency bands resulted in a reduction in DELPHI-EEG performance across all spectral ranges tested (Fig. 4b). The largest decrease in the F1-score was observed following removal of the alpha band (8–12 Hz), with a reduction of 53.04% relative to baseline, followed by reductions of 39.46% for the beta band (12–30 Hz), 34.12% for the slow band (0.5–1 Hz), 33.38% for the delta band (1–4 Hz), and 29.79% for the theta band (4–8 Hz).

Ablation of individual EEG channels resulted in a reduction in DELPHI-EEG performance. The largest decrease in the F1-score was observed following removal of the L2 (left frontoparietal lead), with a reduction of 33.17% relative to baseline, followed by reductions of 31.54% for the R2 (right frontoparietal lead), 22.44% for the R (difference between R1 and R2), 20.65% for the L (difference between L1 and L2), 18.99% for the L1 (left frontal lead), and 12.41% for the R1 (right frontal lead)^24^ (Supplementary Fig. 4a).

The temporal importance plot showed the model put emphasis more on 40-80 seconds within 2-min sample (Supplementary Fig. 4b).

### Subgroup analysis

Subgroup analyses were conducted separately based on the type of anesthesia and the type of surgery. For anesthesia type, the AUROC of the DELPHI-EEG was 0.864 (95% CI: 0.628–0.961) in the total intravenous anesthesia (TIVA) group and 0.872 (95% CI: 0.770–0.943) in the inhalational anesthesia group. For surgical type, the AUROC was 0.899 (95% CI: 0.833–0.981) in the abdominopelvic surgery group, 0.848 (95% CI: 0.689–0.963) in the thoracic surgery group, and 0^25^.867 (95% CI: 0.661–0.960) in the other surgery group (Table 2). The survival analysis showed a significantly different distribution between the predicted positive and negative groups from DELPHI-EEG (*p* < 0.001) (Supplementary Fig. 3a).

**Table 2:** Subgroup analyses of DELPHI-EEG in the 155:1 test set

	*N* (cases)	AUROC [95% CI]	AUPRC [95% CI]	F1 score [95% CI]	Accuracy [95% CI]	Brier score [95% CI]	ICI [95% CI]	Precision [95% CI]	Recall [95% CI]
Anesthesia Type									
TIVA	534	0.864 [0.744–0.959]	0.068 [0.032–0.160]	0.040 [0.017–0.077]	0.554 [0.512–0.592]	0.036 [0.029–0.046]	0.108 [0.097–0.122]	0.021 [0.009–0.040]	0.833 [0.532–1.000]
Inhalational	1806	0.872 [0.770–0.937]	0.033 [0.012–0.097]	0.021 [0.009–0.035]	0.545 [0.526–0.564]	0.029 [0.026–0.033]	0.107 [0.101–0.113]	0.011 [0.005–0.018]	1.000 [1.000–1.000]
Surgery type									
Abdominopelvic	1567	0.899 [0.833–0.981]	0.018 [0.004–0.098]	0.008 [0.003–0.018]	0.534 [0.510–0.552]	0.029 [0.026–0.034]	0.112 [0.106–0.119]	0.004 [0.001–0.009]	1.000 [1.000–1.000]
Thoracic	233	0.848 [0.668–0.957]	0.225 [0.069–0.543]	0.101 [0.037–0.169]	0.464 [0.405–0.522]	0.046 [0.033–0.060]	0.115 [0.104–0.141]	0.053 [0.019–0.093]	0.875 [0.521–1.000]
Others	540	0.867 [0.656–0.949]	0.047 [0.010–0.124]	0.038 [0.010–0.074]	0.624 [0.584–0.668]	0.029 [0.021–0.036]	0.096 [0.089–0.107]	0.019 [0.005–0.039]	1.000 [1.000–1.000]

*AUROC* area under the receiver operating characteristic curve, *AUPRC* area under the precision-recall curve, *ICI* integrated calibration index, *CI* confidence interval, *TIVA* total intravenous anesthesia.

### Sensitivity analysis

Of the 34,550 analyzed cases, 4268 had both the original and CAM-ICU labels. The accuracy and F1-score of the original label to predict the CAM-ICU label were 0.895 and 0.242, respectively. In the testing dataset, the AUROC of the DELPHI-EEG model for predicting the composite outcome was maintained at 0.839 (95% CI: 0.795–0.874, *P* = 0.3707), while the AUPRC increased to 0.123 (95% CI: 0.076–0.198, *P* = 0.007) compared with the performance using the original labels.

## Discussion

This study aimed to develop and validate a deep learning–based intraoperative EEG model for predicting postoperative delirium (POD) and to compare its performance with conventional and machine learning–based approaches. Overall, the DELPHI-EEG showed potentially improved discriminability compared with that of the logistic regression model.

Our model demonstrated a higher AUROC (0.870, 95% CI: 0.789–0.935) than that of a previous ML model using intraoperative frontal EEG signatures and clinical parameters as inputs (AUROC: 0.73–0.80)^18^. Also, our model showed a higher AUROC compared with that of a transformer-based model using time series of intraoperative features, including EEG as inputs (AUROC: 0.772–0.787)^26^. In a previous study, Han et al. developed an ML model incorporating intraoperative EEG features and clinical parameters, which achieved an AUROC of 0.887^19^. However, the model was developed using a manually curated subset of EEG features, which may not be optimal compared to learned features from raw waveform in DELPHI-EEG, and required more than 100 perioperative clinical features as inputs, which may not be feasible in a real-world setting. Moreover, the model by Han et al. was developed specifically for patients undergoing cardiac surgery and included cardiac surgery-specific features, such as intraoperative inotrope use before, during, and after cardiopulmonary bypass (CPB), as well as CPB duration. These features may limit the model’s generalizability to non-cardiac surgical populations. In contrast, DELPHI-EEG was the only model to show a statistically significant improvement in the AUROC relative to the baseline logistic regression model (*p* = 0.004). Other ML models that incorporated age, sex, and EEG features, akin to the Han et al. model, did not demonstrate significant AUROC improvements (XGB, *p* = 0.203; LGB, *p* = 0.181; RF, *p* = 0.246; GB, *p* = 0.598). DELPHI-EEG achieved this superior performance by leveraging a deep learning-based framework to extract spatiotemporal features from raw signals, rather than by relying on manually curated features. By directly processing raw EEG waveforms, DELPHI-EEG extracts multi-scale spatiotemporal representations that reflect both transient and sustained electrophysiologic patterns. This framework enables the identification of POD risk in patients who do not exhibit overt burst suppression or abnormal PSI values, thereby extending predictive coverage beyond traditional indices.

The interpretability analysis revealed that a high risk for POD was correlated with a reduced relative alpha power. This observation is consistent with previous studies where patients with POD had a significantly lower alpha power than the control group in global (0.09 ± 0.06 vs. 0.21 ± 0.08, *p* < 0.0001) and frontal (0.09 ± 0.07 vs. 0.24 ± 0.1, *p* < 0.0001) EEG^27^. In addition, POD was also associated with increased delta and theta power, aligning with a previous report (odds ratio = 1.97; 95% CI = 1.30–2.99) where EEG changes included a greater than 50% increase in delta or theta activity^9^. The generation of intraoperative alpha oscillations has been considered to reflect thalamic hyperpolarization and thalamo-cortical synchronization^28^. Therefore, attenuated alpha power and relatively increased delta and theta power may signal impaired thalamo-cortical connectivity, potentially reflecting age-related neural vulnerability. This link to aging is supported by findings that alpha band power naturally decreases with age (*p* < 0.001)^29^. Given that age was an input variable in the model, attenuated alpha power may represent individual susceptibility to POD^30^.

Frequency-domain perturbation analysis underscored the relative importance of alpha activity in POD prediction, reinforcing its discriminative value compared to other frequency bands. Notably, a prior study reported a > 2-fold increase in alpha power following anesthesia induction in control patients, while it was maintained in those who developed POD^27^. Both groups exhibited significant increases in delta absolute power post-induction^27^. Our ablation analysis showed that any single-frequency band led to significant degradation in model performance, suggesting that while alpha activity holds high predictive value, the DELIPHI-EEG leverages broadband spectral information for robust predictive performance.

Among the analyzed cases, 12.4% had available CAM-ICU data. Although CAM-ICU is widely regarded as the gold standard for delirium assessment in both clinical and research settings, its use is limited to ICU patients. Because our study focused on general surgical patients, many of whom were not admitted to the ICU. Consequently, we established the primary delirium labels based on neuropsychiatric consultations and antipsychotic medication use. However, the sensitivity analysis using the composite outcome revealed that the DELPHI-EEG model’s AUROC was not significantly different from the results with the original labels. The concurrent increase in AUPRC may be due to a higher event rate in the composite standard. This result validates our labeling approach and highlights the model’s generalizability to different annotations.

Our intraoperative EEG model could assist physicians in identifying patients at high risk for POD, allowing for the implementation of targeted prevention strategies. Currently, postoperative dexmedetomidine sedation, multicomponent interventions, and perioperative antipsychotic administration are recognized as effective preventive measures^31^. Given that POD typically occurs several days after surgery under general anesthesia—as reflected by the median onset of 4 days in our cohort—there is a critical window during which close monitoring and rapid interventions could be helpful. Incorporating our model into perioperative care pathways may enhance risk stratification and improve postoperative outcomes, although further prospective validation is warranted to confirm its clinical utility. From a practical perspective, DELPHI-EEG enables real-time application in the operating room by processing continuous EEG waveforms without additional perioperative inputs. Predictions can be updated within milliseconds, introducing no delay beyond the standard EEG acquisition time. Moreover, DELPHI-EEG can be used in conjunction with conventional indices, such as the burst suppression ratio or PSI, to enhance both predictive robustness and interpretability.

Our study has several limitations that should be acknowledged. First, POD was labelled based on psychiatric consultations and administration of delirium-related antipsychotics. This was because assessment and diagnosis data are often missing in electronic health records. However, these medications are commonly considered to manage psychotic symptoms, which occur in approximately 42.7–44.5% of delirium cases^32–34^. In addition, although haloperidol is primarily considered in the management of delirium, other antipsychotics such as quetiapine can be administered for other indications, such as insomnia^35^. Secondly, our analysis did not account for patients who may have developed delirium after hospital discharge. Excluding these cases may have led to an underestimation of the true incidence of postoperative delirium. Third, we developed and validated the DELPHI-EEG model using a single-center cohort with a 6-channel EEG monitoring system. External validation of our model is necessary but may prove challenging due to variations in intraoperative EEG practices across centers, where typically only 2 to 4 frontal channels are used^36^. Moreover, differences in institutional EEG display settings, such as amplitude resolution, can affect signal amplitude and quality, further complicating external validation^37^. Fourth, the burst SR was mechanically calculated by the intraoperative monitor, similar to a previous model that predicted POD using intraoperative EEG suppression, relying on SR analysis rather than manual raw EEG interpretation^38^. Accordingly, expert review may be required, as this approach could influence the accuracy of the model’s performance. Fifth, we did not assess the relationship between DELPHI-EEG predictions and patient frailty, as frailty assessments were not available in the electronic health records. Frailty, a known risk factor for POD, is associated with 1.5- to 2-fold lower intraoperative EEG power across alpha, beta, delta, and theta bands compared to robust patients (all *p* < 0.001)^25^. Therefore, frailty may act as a confounding variable in the observed correlations between DELPHI-EEG predictions and EEG spectral power. Future studies should adjust for frailty when interpreting these correlations, and further analysis is warranted to incorporate frailty as an additional input to the DELPHI-EEG model to improve its predictive accuracy.

In conclusion, the deep learning-based DELPHI-EEG model successfully predicted postoperative delirium using intraoperative EEG waveforms, outperforming conventional suppression ratio-based logistic regression models, although the confidence intervals overlap in AUROC. By identifying attenuated alpha power as a key predictor, the model aligns with established neurophysiological correlates of delirium, while offering a clinically feasible and real-time risk stratification tool. Although external validation across diverse clinical settings is required, DELPHI-EEG shows promise as a clinical tool for POD risk stratification, potentially enabling targeted preventive interventions before delirium onset.

## Methods

### Study design

This retrospective study was approved by the institutional review boards (IRBs) of Seoul National University Hospital (IRB No. 2506-023-1646; approval date: June 10, 2025). Given the retrospective nature of the study, the IRBs waived the requirements for patient consent.

Adults ( ≥18 years) who received surgery under general anesthesia with intraoperative EEG monitoring on Root Platform (Masimo, Irvine, CA, USA) at Seoul National University Hospital (SNUH) between January 2022 and July 2024 were included in this retrospective study. This study followed the Transparent Reporting of a multivariable prediction model for Individual Prognosis Or Diagnosis + Artificial Intelligence (TRIPOD + AI) and the Strengthening the Reporting of Observational Studies in Epidemiology (STROBE) reporting guideline for observational studies^39,40^.

### Study population

Postoperative delirium was defined as cases with (1) newly administered antipsychotics (haloperidol, quetiapine, olanzapine, or risperidone)^41^ or (2) a diagnosis of delirium during the neuropsychiatry consultation after surgery during the same hospital admission. In cases with a neuropsychiatry consultation, the occurrence of a delirium assessment was determined by searching for the keyword “delirium” occurring in the text between the “Assessment” and “Plan” sections of the psychiatry consultation notes in the electronic health records. Patients with no demographic information, preoperative administration of antipsychotics, or a delirium diagnosis were excluded.

Among 34,550 analyzed cases, patients from the extracted cases were randomly divided into a development set and a test set at a ratio of 9:1. The development set was further undersampled at a ratio of 5:1^42,43^. Due to the class imbalance, where the number of POD-positive samples is smaller than that of POD-negative samples, the test set was evaluated using three different random sampling strategies based on unique patients: the original test set, a 1:1 undersampled set, and a 2:1 undersampled set.

### Sample size calculation

To ensure the adequacy of the analyzed sample size, the calculation from a previous study was applied^44^. Using the observed overall prevalence, a target AUROC of 0.80, and prespecified confidence interval widths of 0.10 for AUROC, 0.20 for calibration slope, and 0.20 for observed/expected ratio, the calculation demonstrated that the available sample size was larger than the required minimum size of 30,023 to achieve the desired precision and stability of model performance estimates.

### Data collection

Intraoperative time series of EEG, SR, and PSI were extracted from VitalDB (Seoul National University College of Medicine, Seoul, South Korea). The age, sex, admission and discharge times, operation start and end times, neuropsychiatric consultation timing and content, the CAM-ICU label, and the timing and type of antipsychotics administration of each participant were retrieved from the electronic health records of Seoul National University Hospital.

### EEG preprocessing

For each surgical case, 30 samples of a 2 min EEG waveform were selected by applying a consistent statistical criterion (Supplementary Fig. 5). The mean value was computed from the voltage values at each time point across all 6 EEG channels throughout the whole recording, and a sliding window with a 40 s stride was applied to extract candidate samples. From the candidate samples of each surgical case, the 30 samples with mean values closest to the case-wise mean were retained. This sampling strategy was designed to capture EEG features across various intraoperative phases that are associated with POD, including changes in band power immediately following anesthesia induction, EEG suppression during periods of reduced anesthetic concentration, and alpha spindle activity during emergence from anesthesia^38,45,46^. We excluded samples with any missing values in the 2 min EEG waveform. Samples without SR or PSI values were also excluded. Consequently, some surgical cases included fewer than 30 samples in the final analysis. Samples from surgeries not under general anesthesia were also excluded. Each sample—originally a time series of length 21,378 (corresponding to 2 min at the original sampling rate, 178.15 Hz)—was resampled to a uniform length of 9600 at 80 Hz. A Butterworth bandpass filter between 0.5 and 40 Hz was then applied to remove low-frequency drift and high-frequency noise.

### Deep learning model architecture

An encoding framework comprising a convolutional neural network and a graph convolutional network, followed by a transformer layer, was used in the DELPHI-EEG to predict probabilities for POD^20^. The model input included a 6-channel, 2 min length waveform, age, and sex. The undersampled development set was randomly split according to unique patients into 5-folds for 5-fold cross-validation. The model was trained for each cross-fold with early stopping. The mean training step size of each cross-fold was used as the step size for the training of a final model using the whole undersampled development set.

### Logistic regression and machine learning models

The baseline logistic regression model and ML models were developed using the duration of time where SR > 1%, area under the SR versus time curve where SR > 1%, duration of time where PSI < 25, age, and sex. These variables were selected because they represented the only intraoperative EEG features included in the machine learning model developed by Han et al., thereby allowing for a direct comparison of model performance^19^. The SR values were automatically calculated by the surgical monitoring equipment and subsequently stored using VitalRecorder (Seoul National University College of Medicine, Seoul, South Korea), a software that captures and archives high-resolution biosignal waveforms and vital signs^47^. XGB, LGB, RF, and GB were employed as ML models. The undersampled development set was randomly partitioned by unique patients into 5-folds, and each model was hyperparameter-tuned to optimize the mean AUROC curve across the cross-validation sets. To evaluate the relationship between DELPHI-EEG predictions and EEG burst suppression, we assessed the correlation between model-predicted probabilities and both the duration of time with SR > 1% and the area under the SR-versus-time curve where SR > 1%.

### Statistical analysis

AUROC, AUPRC, F1 score, and accuracy were used to evaluate the discriminative performance of DELPHI-EEG and the logistic regression model. The survival analysis, where survival was defined as remaining free of POD, between the predicted positive and negative labels from DELPHI-EEG for the original test, was performed using the log-rank test. To evaluate the feasibility of DELPHI-EEG for long-term predictions, we assessed the correlation between model-predicted probabilities and the time to POD onset for POD-positive cases in the test set, using the original ratio. The positive label cutoff for model-predicted probabilities was the threshold maximizing the Youden index in the undersampled development set. The calibration performance was evaluated with the Brier score and the integrated calibration index after using the Spline calibration method^48^. DeLong’s test was applied to compare the AUROCs of DELPHI-EEG with those of other machine learning models. Correlations between model-predicted probabilities and relative band powers or time to POD onset were assessed using Spearman’s rank correlation coefficient. Statistical significance was defined as a two-sided *p*-value < 0.05. All model development and statistical analyses were performed using Python 3.9 (Python Software Foundation, Wilmington, DE, USA).

### Interpretability analysis

SHAP analysis was performed post hoc for all ML models (XGB, LGB, RF, GB) to interpret feature contributions and assess the relative importance of SR, PSI, age, and sex.

To assess the interpretability of the DELPHI-EEG model, we analyzed the correlation between its prediction outputs and the spectral characteristics of the raw EEG signals. Specifically, we evaluated the relationship between model-predicted probabilities and the relative band power within the delta (1–4 Hz), theta (4–8 Hz), and alpha (8–12 Hz) frequency bands^27^. For each surgical case, predicted probabilities and corresponding power spectral densities were computed for individual samples with Welch’s method^49^. The resulting density power spectrum was then integrated over frequency via the trapezoidal rule to obtain band-limited power. For each band, relative band power was computed by integrating the power spectral density over the band, summing across channels, and dividing by the power across the 1–40 Hz range.

To assess the spectral contribution of distinct frequency bands to model performance, we implemented a targeted ablation strategy in the frequency domain^50^. Perturbations were applied to each 2 min sample in the original ratio test set. For each frequency band, we performed a discrete Fourier transform using a zero-padded Fast Fourier Transform (FFT). Frequency coefficients corresponding to the targeted band were then replaced with zeros. The modified spectrum was inverse transformed into the time domain using an inverse FFT, producing a perturbed version of the test data with the designated frequency band suppressed. These band-ablated time series were passed through DELPHI-EEG, and predictions were recalibrated using a pre-trained spline calibration model. Binary classification outcomes were generated using a previously determined Youden threshold. The model’s F1-score was then evaluated on the perturbed test data and compared to the baseline F1-score on unperturbed inputs. The percent change in the F1-score following each band-specific perturbation was used to quantify the relative importance of that band in contributing to the model’s decision-making process.

Spatial contributions were assessed by per-channel ablation: for each channel, we set the channel to zero across time, recomputed the calibrated predictions, and evaluated the change in F1 score relative to the baseline. Temporal contributions were assessed with gradient-based saliency. We computed the gradient of the model output with respect to the input, averaging the absolute value of gradients across time to obtain temporal importance profiles.

### Subgroup analysis

Subgroup analyses of DELPHI-EEG were conducted based on anesthesia type (TIVA and inhalational anesthesia), and surgical category (abdominopelvic surgery—including urology, obstetrics, gynecology, and general surgery—thoracic surgery, and other surgical procedures).

### Sensitivity analysis

Among 34,550 analyzed cases, those with both the original label and a CAM-ICU were compared using accuracy and F1-score. Using the testing set, the performance of the DELPHI-EEG for the composite outcome, defined as positive if either the original or the CAM-ICU label was present, was compared to the original label using the paired t-test.

## Supplementary information

Supplementary Information

## Data Availability

The data supporting this study’s findings are available from the corresponding author upon reasonable request.

## Code availability

The code for the preprocessing, training, and inference is available at GitHub (https://github.com/oskumd2/EEG_Delirium).
