# Supplementary material for: Development of a deep learning-based prediction model for postoperative delirium using intraoperative electroencephalogram in adults
Source: NPJ Digit Med. 2025 Nov 17;8:661. doi: 10.1038/s41746-025-02033-y (PMC12623934; doi:10.1038/s41746-025-02033-y)
Supplement: Supplementary file 1 — Supplementary Information [file 41746_2025_2033_MOESM1_ESM.pdf]

Supplementary Table 1. The evaluation metrics of the model for different sampling strategies in the testing dataset.

**Sampling strategy**	**AUROC [95% CI]**	**AUPRC [95% CI]**	**F1 score [95% CI]**	**Accuracy [95% CI]**	**Brier score [95% CI]**	**ICI [95% CI]**	**Precision [95% CI]**	**Recall [95% CI]**
155:1 (original)	0.870 [0.789,0.935]	0.038 [0.017,0.084]	0.026 [0.013,0.040]	0.547 [0.527,0.567]	0.031 [0.028,0.035]	0.107 [0.101,0.113]	0.013 [0.007–0.021]	0.933 [0.778–1.000]
1:1	0.883 [0.741,0.983]	0.915 [0.786,0.989]	0.824 [0.667,0.944]	0.778 [0.630,0.926]	0.281 [0.170,0.398]	0.359 [0.259,0.500]	0.737 [0.529–0.941]	0.933 [0.786–1.000]
2:1	0.927 [0.839,0.990]	0.884 [0.732,0.980]	0.700 [0.500,0.846]	0.755 [0.633,0.878]	0.158 [0.088,0.232]	0.235 [0.160,0.323]	0.560 [0.357–0.759]	0.933 [0.786–1.000]

AUROC, Area under the receiver operating characteristic curve; AUPRC, area under the precision-recall curve; ICI, integrated calibration index; CI, confidence interval

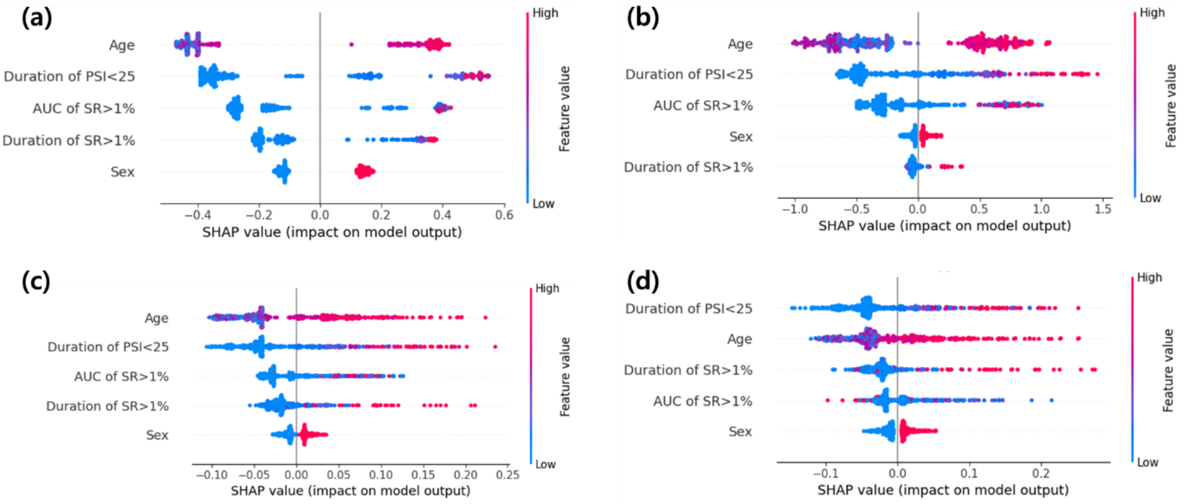

Supplementary Figure 1. Shapley additive explanations plot for test set in 155:1 (original ratio) for machine learning models: (a) XGBoost, (b) LightGBM, (c) Random Forest, and (d) Gradient Boosting Classifier. PSI, Patient State Index; AUC, Area Under the Curve; SR, Suppression Ratio.

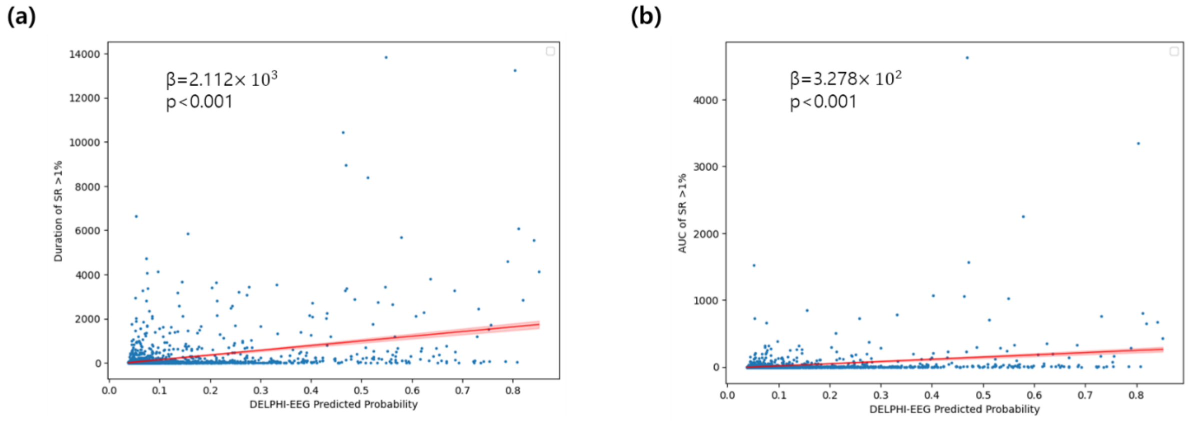

Supplementary Figure 2. Scatter plots showing the relationship between DELPHI-EEG predicted probability and (**a**) the duration of time where SR > 1% and **(b)** the area under the SR versus time curve where SR > 1% in the 155:1 test set. The red line denotes the linear regression fit; the shaded area denotes the 95% confidence interval of the regression estimate. β denotes the slope from Spearman's rank correlation test, and p denotes the corresponding p-value.

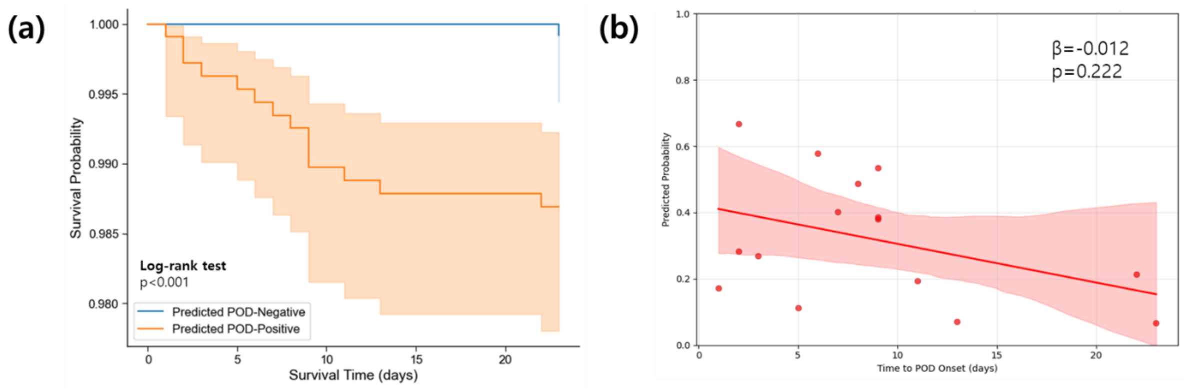

Supplementary Figure 3. (a) The survival curve between the predicted positive and negative labels from DELPHI-EEG for the 155:1 test set, where survival was defined as remaining free of POD. The lines represent the survival curve; the shaded area denotes the 95% confidence interval of the survival probability. (b) A scatter plot showing the relationship between DELPHI-EEG predicted probability and the time to POD onset in the 155:1 test set. The red line denotes the linear regression fit; the shaded area denotes the 95% confidence interval of the regression estimate. β denotes the slope from Spearman's rank correlation test, and p denotes the corresponding p-value.

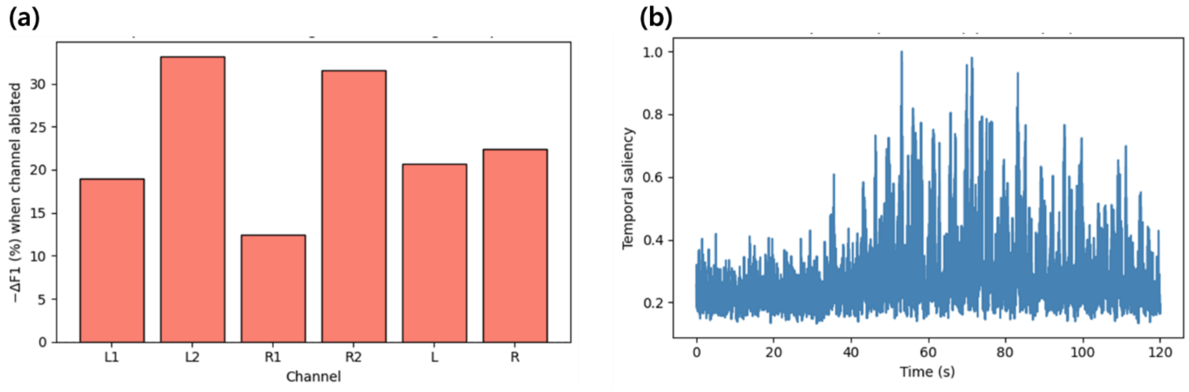

Supplementary Figure 4. (**a**) Percent reduction in F1-score for postoperative delirium (POD) prediction following targeted ablation applied to each 2-min sample. **(b)** The gradient of the model output with respect to the input, averaging |gradients| across time, and is normalized.

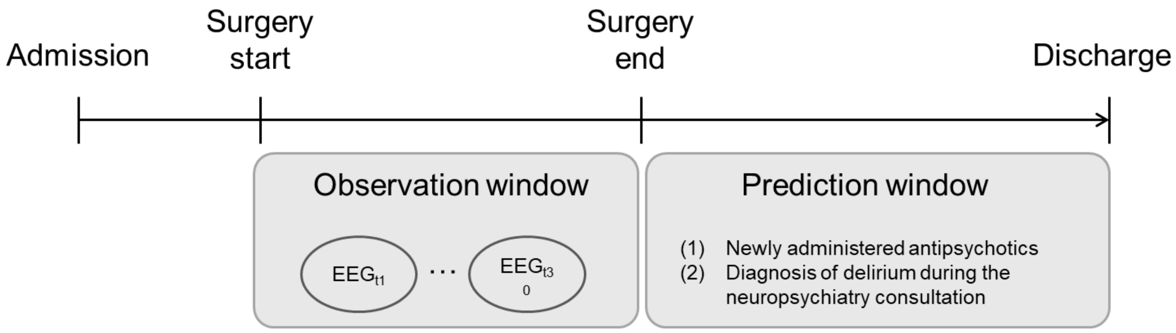

Supplementary Figure 5. Observation and prediction horizons. The DELPHI-EEG model uses 30 samples of the 2-min intraoperative EEG waveforms (EEGt1–EEGt30) selected using a constant statistical criterion. The model predicts postoperative delirium, defined as (1) initiation of antipsychotic medication (haloperidol, quetiapine, olanzapine, or risperidone) or (2) a delirium diagnosis during neuropsychiatry consultation within the same hospital admission.
